# Analysis of Y-P30/Dermcidin expression and properties of the Y-P30 peptide

**DOI:** 10.1186/1756-0500-7-400

**Published:** 2014-06-26

**Authors:** Marina Mikhaylova, Anne Schumacher, Corinna Borutzki, Janine R Neumann, Tamar Macharadze, Tarek El-Mousleh, Petra Wahle, Ana C Zenclussen, Michael R Kreutz

**Affiliations:** 1 RG Neuroplasticity, Leibniz-Institute for Neurobiology, Magdeburg 39118, Germany; 2 Cell Biology, Faculty of Science, Utrecht University, Utrecht, The Netherlands; 3 Department of Experimental Obstetrics and Gynaecology, Medical Faculty, Otto-von-Guericke University, Magdeburg 39120, Germany; 4 Developmental Neurobiology, Faculty of Biology and Biotechnology, Ruhr University, Bochum, Germany

**Keywords:** Placenta, Syndecans, Dermcidin Y-P30, PBMCs

## Abstract

**Background:**

The survival promoting peptide Y-P30 has a variety of neuritogenic and neuroprotective effects *in vitro* and *in vivo*. In previous work we reported the expression of Y-P30/dermcidin in maternal peripheral blood mononuclear cells (PBMCs) and the transport of the protein to the fetal brain. In this study we analyzed hormonal regulation of Y-P30 in human immune cells and expression of Y-P30 in the placenta. We further studied the stability and secretion of the Y-P30 peptide.

**Results:**

We found indications that Y-P30 might be produced in human placenta. The Y-P30 mRNA was rarely found in isolated human PBMCs and alpha-feto-protein, human chorionic gonadotropin as well as estradiol combined with progesterone could not induce Y-P30 expression. Y-P30 was found to be extraordinarily stable; therefore, contamination with the peptide and the Y-P30/Dermcidin precursor mRNA is a serious concern in experiments looking at the expression of Y-P30/Dermcidin. In cultured cell lines and primary neurons we found that Y-P30 could be released, but neuronal uptake of Y-P30 was not observed.

**Conclusions:**

Our data suggest that a source of Y-P30 apart from eccrine glands might be the placenta. The peptide can be secreted together with the signaling peptide and it might reach the fetal brain where it can exert its neuritogenic functions by binding to neuronal membranes.

## Background

**Figure 1:**
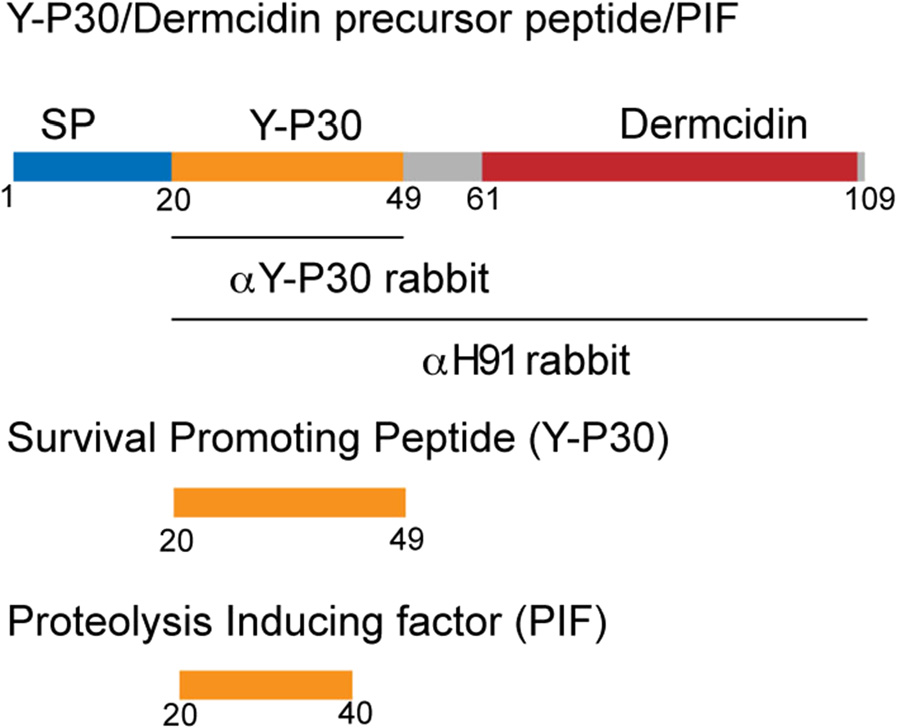
**Schematic of the Y-P30/dermcidin precursor.** SP: signal peptide. PIF: proteolysis inducing factor.

The survival promoting peptide Y-P30 has neurite outgrowth promoting effects *in vitro*[1,2]. In addition potent neuroprotective effects against various insults including traumatic brain injury, oxidative stress, hypoxia and optic nerve crush have been ascribed to the peptide [3-6]. The underlying mechanisms for this broad spectrum of biological activities are still largely unclear. Cell migration and neurite outgrowth promoting activities appear to rely on binding of Y-P30 to syndecans and pleiotrophin [2,7-9]. Other interactions have been reported for calreticulin [4], HSP70 [10] and NCK [11] and it was speculated that these interactions trigger the anti-apoptotic effects of the peptide. Y-P30 is the N-terminal portion of a larger propeptide precursor that encodes a second bioactive peptide, dermcidin (Figure 1), which is secreted by human sweat glands and has been shown to play an important role in antimicrobial defense [12]. The identity of peptide fragments cleaved from the N-terminus of the precursor protein is not clear. The sequence of Y-P30 overlaps with that of the proteolysis inducing factor [13,14]. In contrast to Y-P30, PIF is glycosylated and was originally identified as a factor that induces cachexia in mice and cancer patients [13], although this role in humans has been disputed [15].

Our previous work has suggested that Y-P30 could be a blood-borne factor in rats which is produced by PBMCs of the maternal immune system [1]. However, despite the identification of several Y-P30/dermcidin peptides with mass-spectroscopy in various mammals, the dermcidin gene seems to be absent in the genome of non-primate species [16,17]. Therefore, these previous findings in rodents raise questions regarding the source of the peptide. In human, a prominent expression in eccrine sweat glands is not disputed; further, some studies reported the Y-P30/dermcidin precursor protein in human blood in acute ischemic heart disease [18,19] and in placenta [20]. Other studies found the Y-P30 mRNA in human brain [21] and neural cell lines [3]. Moreover, dermcidin has been reported a candidate oncogene in human cancer [21,22] and to promote proliferation of mouse, rat and human tumor cells [13,22-24], but see also [15]. In this study we aimed to address open questions and conflicting results regarding the expression, secretion and source of Y-P30.

## Methods

### cDNA constructs

**Figure 2:**
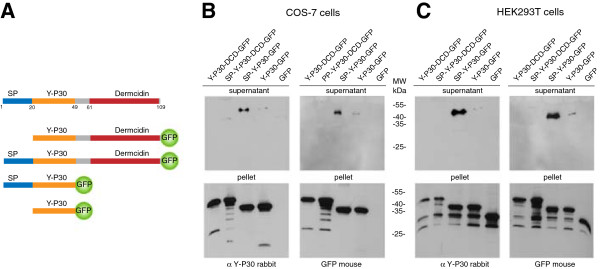
**Exocytosis and processing of the Y-P30/dermcidin precursor. (A)** Schematic of the constructs employed. **(B + C)** GFP-fusion constructs depicted in A were transfected into COS-7 and HEK-293 T cells. 24 hours after transfection the culture medium (supernatant) was collected and precipitated with 100% acetone to increase protein concentration. Cells were washed, harvested and lysed (pellet). Both, cell pellet and supernatant were analyzed by immunoblotting with anti-Y-P30 or anti-GFP antibodies. SP: signal peptide.

cDNA constructs (see Figure 2) encoding the human Y-P30-Dermcidin (DCD) precursor (aa 1–109), Y-P30-Dermcidin precursor lacking the N-terminal signal peptide (aa 21–109), Y-P30 with (aa 1–49) and without (aa 21–49) signal peptide were subcloned into the pEGFP-N1 vector (Clontech). pEGFP-N1 was used as a negative control. For bacterial expression, Y-P30 containing the signal peptide (aa 1–49) was subcloned into a pTYB21 vector. Expression and purification of Y-P30 via the Intein system was performed according to manufacturer’s instructions (Impact kit, NEB) and previously published protocols [25].

### Fingerprint and in vitro stability assay

Probands were asked to keep washed and air-dried hands for 30 min in a plastic bag and then to press the thumb gently on a piece of nitrocellulose (BA85, Whatman). The membranes were kept for up to 5 days at RT on a lab shelve in a standard plastic cell culture plate. The staining was not compromized after prolonged storage. For staining, the membrane was soaked in TBS, blocked with 4% BSA in TBS, incubated overnight at 8°C in the primary antibodies against DCD (H-91 rabbit anti-DCD, 1:50; Santa Cruz) and Y-P30 (N20 goat anti-DCD N-terminal peptide, 1:50; Santa Cruz), washed in TBS with 0.05% Tween-20, incubated with appropriate alkaline phosphatase-conjugated secondaries for 1 h, followed by alkaline color reaction with NBT-BCIP. Membranes were dried and photographed.

Y-P30 (aa 21–49) was synthesized as described previously [1]. For measuring stability of the peptide in vitro, Y-P30 was diluted in PBS (2 μg/μl) and incubated at room temperature over 24, 48 and 72 hours as well as 7 and 14 days. Additionally, the effect of different denaturing agents (urea, 8 M; dithiothreitol (DTT), 50 mM and 100 mM; iodoacetamide, 50 mM) was studied with incubation times of 2 and 12 hours. Thereafter, 4x sample loading dye (containing 5% beta-mercaptoethanol and 8% SDS) was added to 1x final concentration, all samples were boiled for 5 min at 95°C and subjected to SDS-PAGE with subsequent immunoblotting using a rabbit anti-Y-P30 antibody described previously [1,2].

### Cleavage assay

Cleavage of the Y-P30-Dermcidin precursor in COS-7 cells (monkey) and HEK-293 T cells (human) was studied by transfecting cells with different Y-P30-Dermcidin-GFP fusion constructs. Transfection of cells was done as described previously [26]. Briefly, cells were seeded on 6-well plates supplemented with DMEM and used for transfection before they reached confluence. Transfection was performed using Lipofectamine (Invitrogen) according to the supplier’s manual. 48 hours after transfection the medium was collected. Cells were harvested in 1 x PBS, washed and lysed in 10 mM Tris buffer. Proteins samples from the medium and cell lysates were subjected to SDS-PAGE and immunoblotting with an rabbit anti-Y-P30 and mouse anti-GFP antibody.

### ELISA test

To quantitatively assess the level Y-P30/dermcidin precursor in placenta extract, plasma and serum from pregnant women, an ELISA kit for dermcidin (Uscn Life Science) was used according to the manufacturer’s protocol. Briefly, placenta samples were homogenized in PBS and treated with 0.5% (final concentration) Triton X-100. The Triton X-100 extracts were used for ELISA. Plasma and serum proteins were enriched by precipitation with 100% ethanol at -20°C over 72 h, and precipitates were re-suspended in PBS.

### Y-P30 supplementation assay

Y-P30-GFP was produced by *in vitro* ligation of Y-P30 containing the signal peptide and cys-GFP in the presence of 10 mM MESNA via thiol-mediated protein ligation. After 24 h incubation at 4°C the sample was dialyzed into PBS buffer, aliquoted and stored at -80°C. Differentiated primary cortical neurons were supplemented with Y-P30-GFP (10 μg/1 ml) for 24 h, then fixed and stained as described below.

### Co-culture of primary neurons and HEK-293 T cells

Primary cortical neurons were plated on 18 mm coverslips as described previously [27]. HEK-293 T cells were plated on 6-well plates and transfected with the various Y-P30-Dermcidin-GFP plasmids or empty GFP control plasmids using the calcium phosphate method. 24 h after transfection, supplemented DMEM medium was removed and HEK-293 T cells were gently re-suspended in 500 μl of neurobasal medium with antibiotics, glutamine and serum. Then, 30 μl of HEK-293 T cell suspension was added to DIV1 primary neurons. Cells were grown in co-culture for the next 24 h, then fixed and stained as described below.

### Immunocytochemistry, laser scanning microscopy and image analysis

Coverslips with cortical neurons supplemented with Y-P30-GFP, or cortical neurons co-cultured with transfected HEK-293 T cells were fixed with 4% PFA for 10 min at 37°C, extensively washed with PBS and immunostained for the neuronal marker MAP2 (1:1000; mouse, Sigma) and Y-P30 (rabbit, 1:100) as described before [28]. Fluorescence images were obtained on a TCS SP5 II confocal laser scanning microscope (Leica, Germany) using a 63x oil objective. Images were acquired as z-stacks with 0,3 μm z-step. Maximum projections of z-stack were created in the ImageJ program (ImageJ, NIH).

### Sample collection

Human blood samples were obtained from non-pregnant women or healthy pregnant women at all three trimesters. PBMCs were isolated by density gradient centrifugation and washed twice in PBS. Then, PBMCs were either used freshly for hormonal stimulation or frozen as cell pellet at -80°C for Y-P30/dermcidin expression analysis. Additionally, placenta tissue samples were obtained from patients either suffering from spontaneous abortions or pre-eclampsia. Placenta samples from normal pregnant women undergoing elective termination of pregnancy during their first trimester (labeled as interuptio in the Figures) or having normally progressing pregnancies until birth (labeled as TERM in the Figures) were taken as controls. Placental tissue was immediately snap frozen and stored at -80°C. All women provided informed consent and tissue sampling was approved by the Ethics Board of the University of Magdeburg (study 28/08). The characteristics of the patients included in the study are shown in Table 1.

**Table 1:** Characteristics of Normal Pregnant (NP), Spontaneous Abortion (SA) and Pre-Eclamptic (PE) patients at different pregnancy stages

***PBMCs***	***Trimester***	***Age***	***Week of pregnancy***
NP (n = 16)	I	26,25 ± 7,30	10,19 ± 1,98
NP (n = 2)	II	28,50 ± 0,50	18,5 ± 3,5
NP (n = 6)	III	30,33 ± 3,64	30,33 ± 4,15
***Placental tissue***	***Trimester***	***Age***	***Week of pregnancy***
NP (n = 8)	I	32,75 ± 6,32	9,88 ± 1,17
SA (n = 12)	I	33,18 ± 3,86	10,13 ± 3,28
NP (n = 12)	III	30,55 ± 5,19	39,33 ± 1,11
PE (n = 8)	III	35,50 ± 5,32	37,33 ± 1,25

Data are presented as mean plus standard deviation. No statistical differences could be detected among groups.

### Hormonal stimulation of PBMCs

5 × 10^6^ PBMCs from non-pregnant women were first cultured for 24 h in RPMI 1640 medium (Invitrogen, Karlsruhe, Germany) supplemented with 100 ng/ml penicillin/streptomycin and 3% of charcolized fetal bovine serum (FBS) (Biochrom, Berlin, Germany) to reduce undesirable side effects due to hormonal contaminations of FBS. Afterwards, the cells were stimulated for 1 h with 50 μg/ml alpha-feto-protein (Antikoerper-Online, Aachen, Germany), 100 IU/ml human chorionic gonadotropin (Pregnyl, Organon, Netherlands), 10 ng/ml progesterone (Sigma, Steinheim, Germany), 100 pg/ml estrogen (Sigma, Steinheim, Germany), or progesterone and estrogen in combination. PBMCs cultured alone served as controls. After stimulation PBMCs were harvested, washed twice in PBS and frozen as cell pellets for Y-P30/dermcidin expression analysis by PCR.

### RNA isolation and cDNA synthesis

Total RNA was isolated from hormonal-treated and non-treated PBMCs as well as from placenta tissue samples as described elsewhere [29] and converted into cDNA for PCR analysis. Briefly, PBMCs and placental tissue (100 mg) were resolved in 1 ml Trizol® Reagent (Invitrogen, Darmstadt, Germany) and tissue samples were further disaggregated using a homogenizer (Ultra Turrax T8; Ika, Germany). The RNA was then extracted with 200 μl chloroform (Sigma, Steinheim, Germany), precipitated with isopropanol (Roth, Karlsruhe, Germany), washed with 80% ethanol (Otto Fischer, Magdeburg, Germany) and finally re-suspended in RNAse-free water (Berlin Chemie, Berlin, Germany). RNA concentration was determined by reading ultraviolet absorbance at 260 nm.

To obtain cDNA, 2 μg of total RNA were diluted in RNase-free water and added with oligo-dT primer (Promega, Mannheim, Germany). After incubation at 75°C for 10 min, samples were placed on ice, and dNTP (2.5 mM; Pharmacia, Freiburg, Germany), DNase I (2 U/ml; Stratagene, Amsterdam, Netherlands) and RNase inhibitor (40 U/ml; Promega, Mannheim, Germany) mixed in reaction buffer were added. The reaction mix was incubated for 30 min at 37°C and heated to 75°C for 5 min. The addition of the reverse transcriptase (200 U/ml; Amersham) and RNase inhibitor started the reverse transcription. The reaction mix was incubated at 42°C for 60 min followed by inactivation of the enzymes at 94°C for 5 min. cDNA was stored at -20°C until use.

### Nested polymerase chain reaction (PCR)

Nested PCRs intend to reduce non-specific primer binding and are carried out in two successive PCR runs, involving two sets of primers. For the first PCR run, the amplification reaction (24 μl) consisted of 2 μl cDNA, 5 μl 5x Green GoTaq® Reaction Buffer, 0.5 μl dNTP mix (10 mM), 0.125 μl GoTaq® DNA polymerase (all from Promega, Mannheim, Germany), 0.5 μl of the forward primer (GGG AAT TCA TGA GGT TCA TGA CTC TCC TCT), 0.5 μl of the reverse primer (ACG CGC CGA CTC ACT ATA GTA CTG AGT CAA) and filled up to 24 μl with double distilled water. No template controls (NTCs) containing water instead of cDNA were included. The Y-P30/dermcidin precursor cDNA was used as a positive control. For the second PCR run, the reaction mix (20 μl) contained 5 μl PCR product from the first run, 5 μl 5x Green GoTaq® Reaction Buffer, 0.5 μl dNTP mix (10 mM), 0.1 μl GoTaq® DNA polymerase, 0.5 μl of the forward primer (AGC ATG AGG TTC ATG ACT CTC), 0.5 μl of the reverse primer (CAC GCT TTC TAG ATC TTC GAC) and double distilled water up to 20 μl. PCR reactions were performed as follows: initial denaturation step of 2 min at 95°C, followed by 45 sec at 95°C, 45 sec at 58°C (first run) or 56°C (second run) and 1 min at 72°C for 40 cycles. Additionally, the *house-keeping* gene HPRT was amplified in all samples. Subsequently, PCR products were sequenced. PCR products were visualized by agarose gel electrophoresis (2% gel, 100 V for 2.5 h).

### Quantitative (q) RT-Real-time PCR

For RT-PCR analysis, TaqMan (for the Y-P30/dermcidin transcript) as well as SYBR® Green (for the syndecan-3 and -4 transcripts) technology were conducted using an iQ™5 Multicolor Real-Time PCR detection system (Bio-Rad, Munich, Germany). For Y-P30/dermcidin, amplification reactions (13 μl) consisted of 1 μl of cDNA, 6.25 μl of mastermix containing PCR buffer, dNTPs, MgCL_2_, and Ampli-*Taq* DNA polymerase (Eurogentec, Berlin, Germany), 3 μl of the primer/probe mix (Hs01556561_g1 and Hs01556562_g1; Applied Biosystems, Darmstadt, Germany), and 2.25 μl of water. No template controls, NTCs, containing water instead of cDNA were included. All reactions were performed in duplicates as follows: initial denaturation step of 10 min at 95°C, followed by 10 sec at 95°C and 1 min 30 sec at 60°C for 40 cycles.

For syndecan SDC-3 and SDC-4 detection in placenta samples, amplification reactions (13 μl) consisted of 1 μl of cDNA, 6.5 μl of Power SYBR® Green mastermix containing PCR buffer, dNTPs, MgCL_2_, and Ampli-*Taq* DNA polymerase (Life Technologies, Darmstadt, Germany), 3 μl of the primer mix (SDC-3: Fwd: CGA TGA TGA ACT GGA TGA CCT C, Rev: CTG TCT CAA TGC CCG ACT, SDC-4: Fwd: CAG ACG ATG AGG ATG TAG TGG, Rev: GGA TGG ACA ACT TCA GGG C), 0.5 μl fluorescein and 2 μl of water. NTCs containing water instead of cDNA were included. All reactions were performed in duplicates as follows: initial denaturation step of 10 min at 95°C, followed by 30 sec at 95°C, 45 sec at 58°C and 30 sec at 72°C for 40 cycles. Fluorescence, and thus quantity of PCR product, was continuously monitored by iQ5 software (version 2.0 for Windows, Bio-Rad, Munich, Germany). All samples were normalized to their β-actin content. Relative expression was calculated using the formula 2^-ΔCT^ (CT; cycle threshold), where ΔCT is (gene of interest CT)–(reference gene CT).

### Protein isolation and Western blot analysis

For detection of SDC-3, SDC-4 and Y-P30/dermcidin protein expression, proteins were isolated from frozen placental tissue pieces as described previously [30] and their concentration was assessed by using the BCA Protein Assay (Thermo Fischer Scientific, Bonn, Germany). For Western blot analysis proteins were separated on a 10% (SDCs) polyacrylamide gel under denaturizing conditions. After electrophoresis proteins were transferred to PVDF membranes. Membranes were incubated overnight with the following primary antibodies: polyclonal goat anti-human SDC-1/3 or polyclonal rabbit anti-human SDC-2/4 (all from Santa Cruz, Heidelberg, Germany) and rabbit anti-Y-P30 antibody [1]. After three washing steps with TBST (TBS with 0.05% Tween) for 5 min each, the membranes were incubated with an HRP-conjugated anti-rabbit (Thermo Fisher Scientific, Bonn, Germany) or biotin-conjugated anti-goat (Dako, Glostrup, Denmark) antibody diluted 1:500 for 60 min at RT, followed by an avidin/horseradish peroxidase complex (Vector Laboratories, Burlinghame, USA). The ß-actin (Sigma, Steinheim, Germany) served as loading control. The chemiluminescence signal was generated by using luminol (Sigma, Steinheim, Germany), 4-hydroxycinnamic acid (p-coumaric acid; Sigma, Steinheim, Germany), and hydrogen peroxide (Merck, Darmstadt, Germany). The intensity of the bands was quantified by using the GeneSnap® Software, Version 4.01c from Syngene.

### Statistical analysis

Data obtained for protein and mRNA expression is presented as medians in graphs showing individual values for each animal. Analysis of statistical differences among the groups was performed using the nonparametric Kruskal-Wallis test. For analyzing differences between two particular groups the Mann Whitney-*U*-test was applied. In all cases, p < 0.05 was considered to be statistical significant.

## Results

### qPCR to analyze the presence of Y-P30/dermcidin transcript in human PBMCs yields variable results

Based on previous work [1] we first set out to analyze the presence of Y-P30/dermcidin mRNA in human blood cells from pregnant and non-pregnant women. We purified PBMCs and analyzed 24 samples from pregnant women at different pregnancy stages (16 from the first trimester, 2 from the second trimester and 6 from the third trimester) and performed nested PCR as well as RT-PCR with Y-P30/dermcidin specific primers. Of the 24 PBMC samples of pregnant women, only two from the first trimester showed a positive result by nested PCR, and one sample each from the second and third trimester was positive for Y-P30/dermcidin expression by RT-PCR (Table 2). Thus, analysis of Y-P30/dermcidin mRNA expression in a larger sample than we published earlier [1] gave in most cases negative results. Moreover, attempts to identify a subpopulation of PBMCs that express the Y-P30/dermcidin mRNA failed (data not shown). However, the positive results have to be treated with caution. Previous work has shown that the mRNA Y-P30/dermcidin is extremely stable and can be used in forensic medicine for sweat detection [31]. It was also reported that skin and sweat contaminations result in a transfer of Y-P30/dermcidin mRNA to clothes, which is still detectable several days later [31]. Even a handkerchief used to wipe the eye brow several times within 12 h was contaminated with Y-P30/dermcidin mRNA [31]. One therefore has to take into account the possibility of contaminations: trace amounts of skin-derived transcripts e.g. from the experimenter might deliver a positive readout when using very sensitive PCR methods.

**Table 2:** Presence of Y-P30/dermcidin transcript in human PBMCs at different pregnancy stages

***PBMCs***	***Trimester***	***Positive for Y-P30/dermcidin transcript by nested PCR/samples tested***	***Positive for Y-P30/dermcidin transcript by RT-PCR/samples tested***
NP (n = 16)	I	2/16	2/9
NP (n = 2)	II	0/2	1/1
NP (n = 6)	III	0/6	1/3

**Figure 3:**
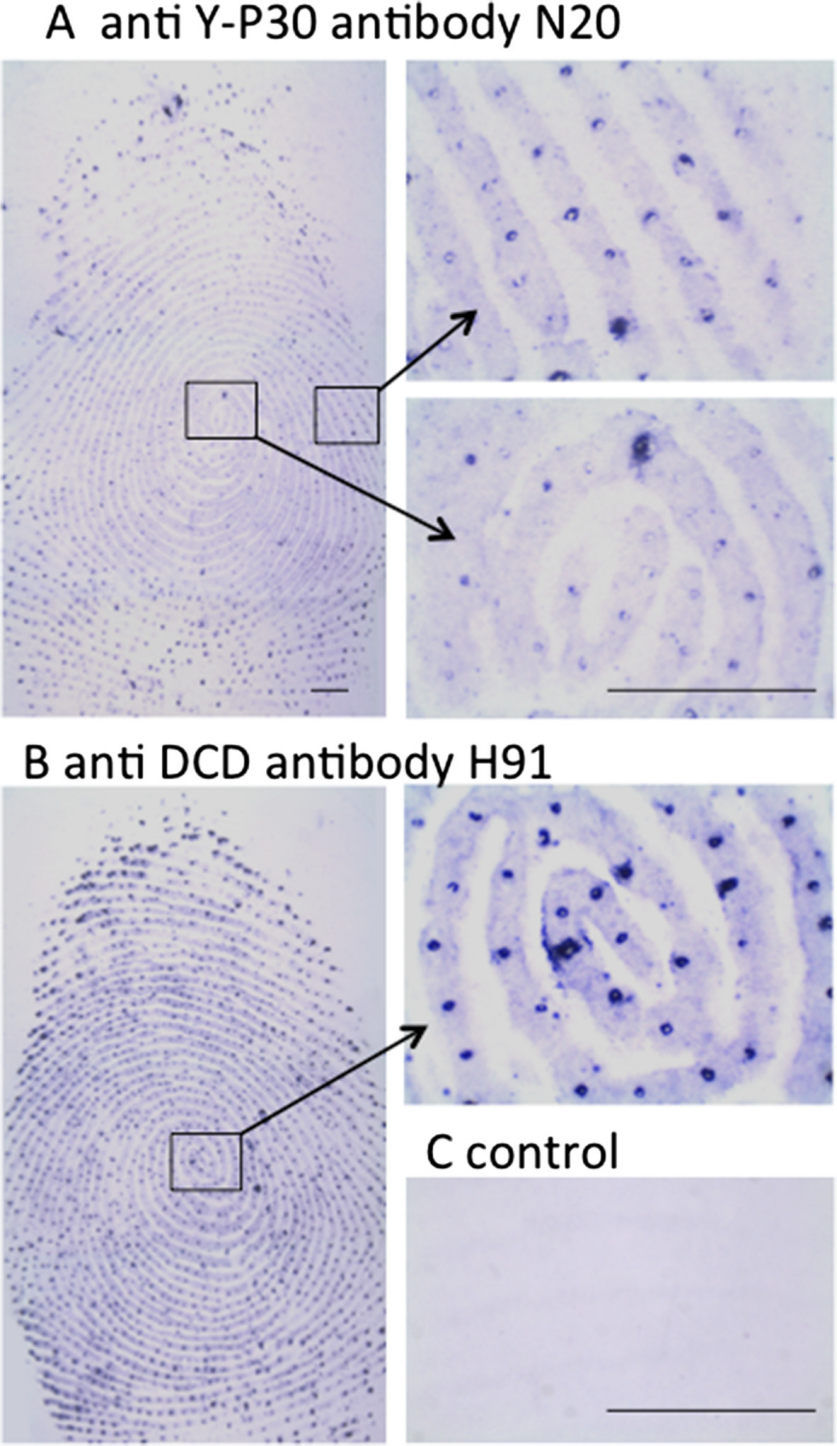
**Y-P30 and dermcidin immunoreactivity is present on human fingerprints. (A + B)** Human fingerprints on BA85 nitrocellulose were stained 2 days after sampling with antibodies against DCD and Y-P30 in **A** and **B**, respectively. Immunoreactive material concentrates at the pores of sweat glands aligned in epidermal ridges. **C**, fingerprint stained under ommision of the primary antibody against Y-P30. Scale bars = 1 mm.

Similar contamination problems might be encountered with the Y-P30 and dermcidin peptide. Studies have shown that the peptide can be exhaled in cancer patients [32], asthma, and also healthy volunteers [18], and is abundantly present on fingerprints [33]. We could replicate these findings and found intense Y-P30/dermcidin immunoreactivity with two different antibodies on human fingerprints, and the antigenicity of the skin-derived peptides on filter paper was stable for several days (Figure 3).

**Figure 4:**
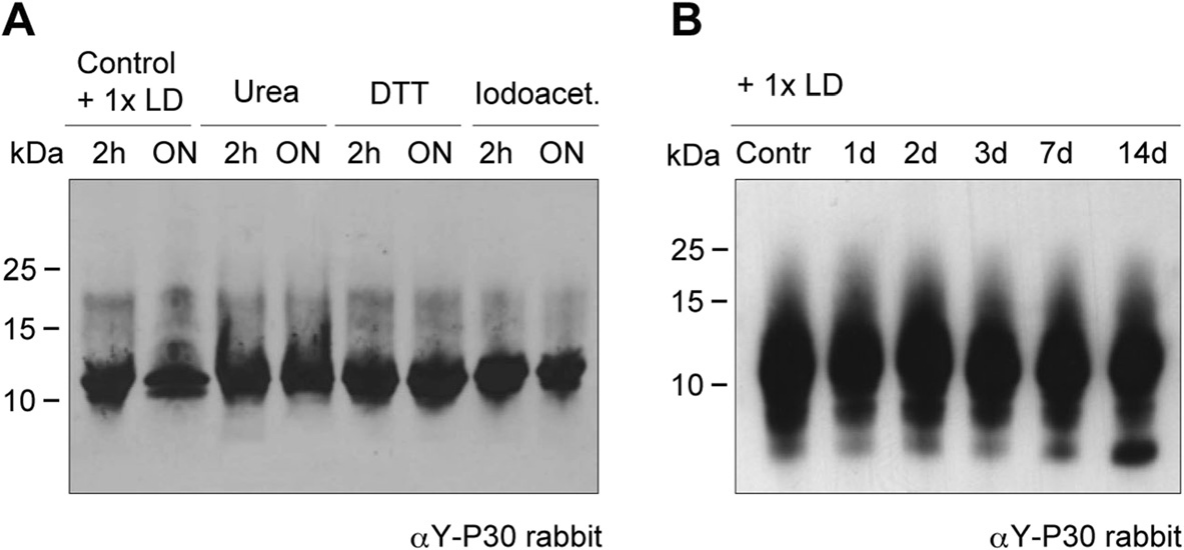
**The Y-P30 peptide is very stable and forms oligomers that migrate at a molecular weight between 10–15 kDa. (A)** Oligomers are highly stable and resistant to SDS and reducing agents added for 2 h or overnight (ON). 10 μg synthetic peptide were loaded in each lane. **(B)** Storage of the peptide in solution for up to 14 days at room temperature in an Eppendorf tube on a lab bench does not lead to obvious degradation of the peptide multimer. 13,5 μg of the peptide were loaded in each lane.

In previous work we have shown oligomerization of the Y-P30 peptide [2], and that oligomers are able to cluster pleiotrophin of the extracellular matrix and neuronal membrane-bound syndecans, which are important for neuronal signaling [2] and the induction of axonal growth [8,9]. We followed up on these results and performed additional oligomerization and stability assays with the synthethic Y-P30 peptide under various conditions (Figure 4). As in previous work we found SDS-resistant multimerization of the synthetic Y-P30 peptide in the range of 10–15 kDa (Figure 4A). The 10–15 kDa oligomer species now turned out to be resistant to urea, DTT and iodoacetamide inclusion in the loading buffer (Figure 4A). Moreover, we found that the 15 kDA oligomer is stable for at least 14 days. No apparent degradation was detectable in samples previously kept from 1 to 14 days at room temperature in an Eppendorf tube on a bench shelf with regular light exposure (Figure 4B). Thus, the Y-P30 peptide is extremely stable suggesting that even minor contaminations might last for longer periods of time.

### Y-P30 and dermcidin are expressed in human placentas of first trimester pregnancies

**Figure 5:**
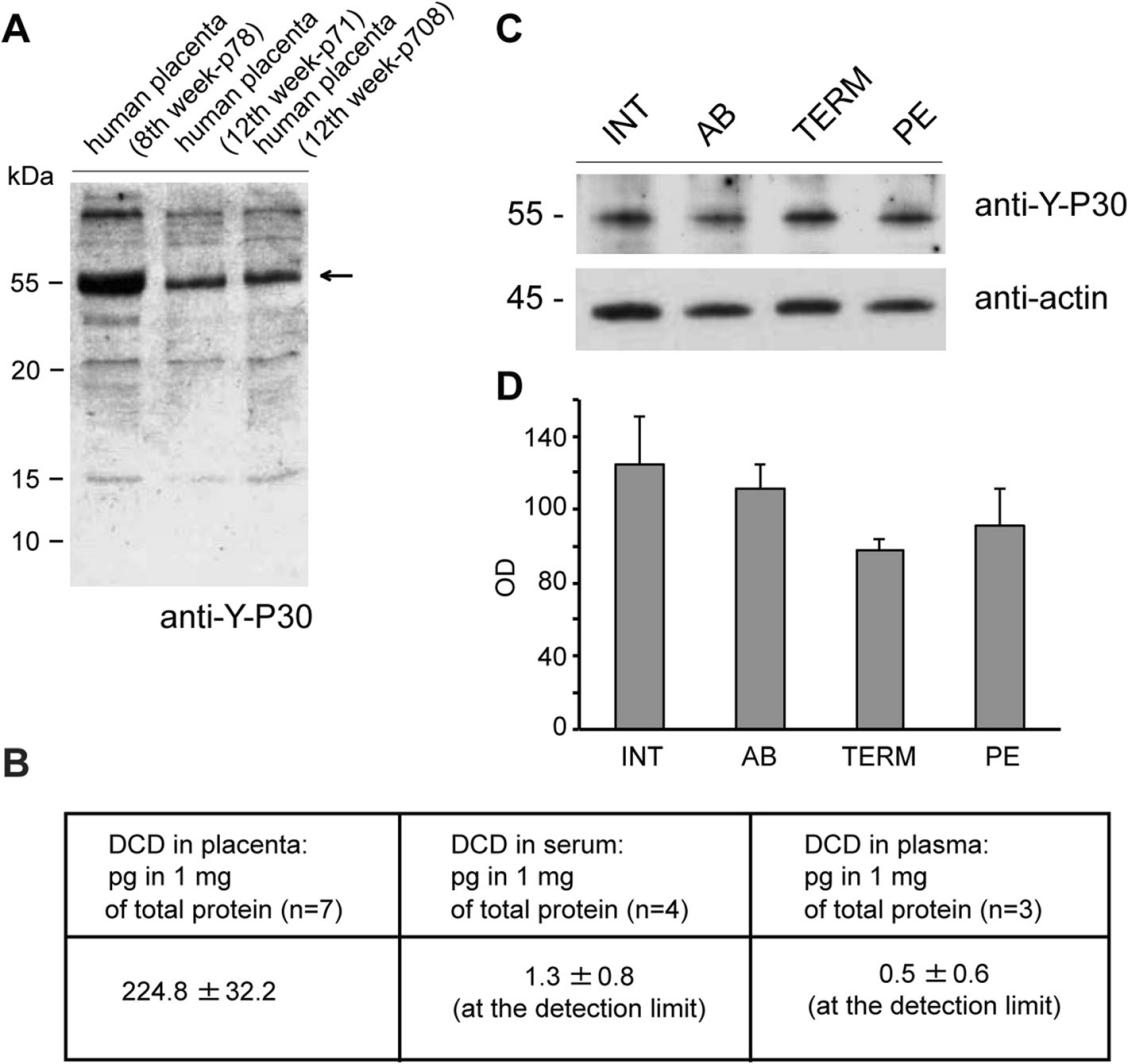
**Western blot analysis. (A)** The Y-P30/dermcidin precursor peptide is present in placenta at a molecular weight above 50 kDa (arrow). **(B)** With an ELISA assay we could also detect dermcidin in placenta extracts. Dermcidin levels in serum and plasma from the same patients were at the detection limit. **(C + D)** Y-P30/dermcidin precursor expression in placenta from normal pregnant women (INT, interruptio; TERM, after birth) and women suffering from pregnancy complications (SA, spontaneous abortion; PE, pre-eclampsia).

We next followed up on previous reports documenting the expression of Y-P30 and dermcidin in placenta [20]. We studied this in human samples and found that in fact Y-P30/dermcidin mRNA was present in human placenta from the first trimester of normal pregnancies (n = 8; data not shown). Given the potential pitfalls outlined above with mRNA detection, we next analyzed in lysates of human placenta the presence of Y-P30/dermcidin protein by means of an antibody recognizing the larger precursor protein containing both, Y-P30 and dermcidin (Figure 5A). We observed the presence of the precursor protein (Figure 5A) at two different time points, week 8 and week 12 of pregnancy. There is conflicting evidence on the size of the Y-P30/Dermcidin precursor in immunoblotting. Our data are in accordance with previous studies [3,10,11,13], who also reported higher molecular weight bands above 50 kDa when run on a standard reducing SDS-polyacrylamide gel. Besides oligomerization, glycosylation and albumin-binding can modify the peptide in tissue and could possibly account for these higher MW bands [3,10,13]. Further, using a dermcidin ELISA assay, we could also confirm the presence of dermcidin in placenta extracts (Figure 5B). The levels of dermcidin in serum and plasma obtained from the same patients were at the detection limit (Figure 5B). We observed no lower Y-P30/dermcidin levels in patients suffering from pre-eclampsia as compared to samples from normally developing pregnancies (Figure 5C + D). In addition, administration of the peptide at doses of 100 μg, 250 μg and 500 μg did not induce pre-eclampsia in mice (unpublished observations) and had also no effect on different immune cell populations (unpublished observations).

Dermcidin seems to be regulated by various stimuli [34]. To test the hypothesis that hormones synthesized by the placenta and released to the circulation might evoke an expression of Y-P30 in cells of the immune system (PBMCs), we studied the effect of human chorionic gonadotropin (hCG), estradiol, progesterone and the combination of the latter two. The effect of alpha-feto-protein (αFP), which is released by the fetus into the maternal circulation, was also analyzed. We found that estradiol and progesterone at natural amounts present in the serum during first trimester as well as the combination of both hormones did not provoke the expression of Y-P30 in PBMCs from non-pregnant donors (data not shown). In addition, also αFP and hCG did not induce Y-P30 expression (data not shown). Thus, molecules produced by the placenta or the fetus during early pregnancy seem not responsible for the occasional presence of Y-P30 transcripts in immune cells.

### Y-P30 binding proteins are expressed in placenta

**Figure 6:**
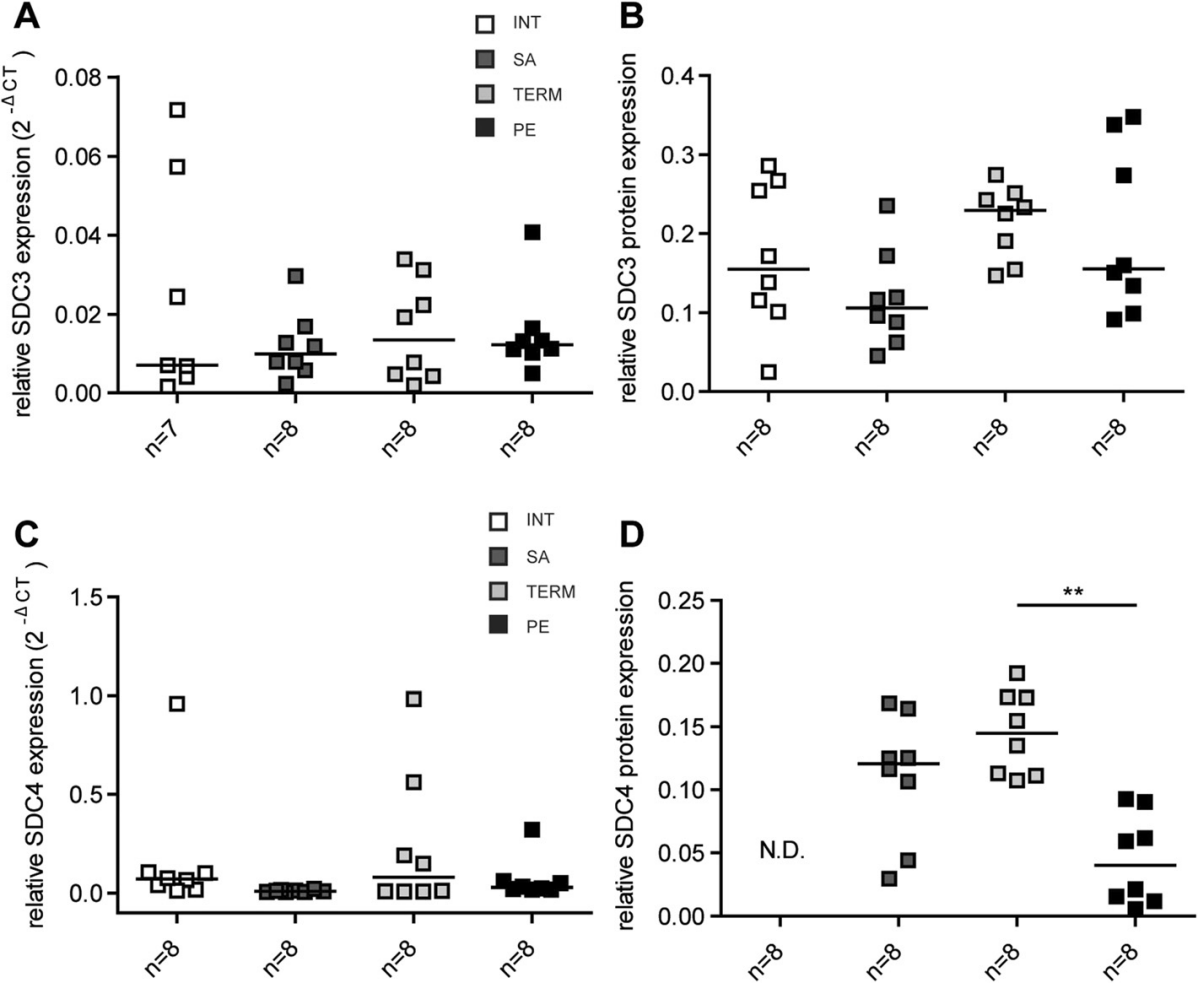
**Syndecan-3 and -4 expression in placenta. (A + C)** mRNA expression, **(B + D)** Syndecan-3 and -4 protein expression. qPCR and Western blot analysis show differential expression patterns of syndecan-3 and -4 in human placenta samples from normal pregnant women (INT, interruptio; TERM, normal pregnancy, tissue sampled after birth) and women suffering from pregnancy complications (SA, spontaneous abortion; PE, pre-eclampsia). mRNA and proteins were isolated from snap frozen placenta samples. Expression was normalized to the house-keeping gene beta-actin. Each square represents one sample and data are presented as medians. Statistical differences between two groups were analyzed by the Mann–Whitney *U*-test. N.D., non-detectable. **p ≤ 0.01.

Since Y-P30 can be detected in placenta, we next investigated whether its binding proteins, syndecans [2] are expressed in the placenta as well. We indeed found that, syndecan-3 and syndecan-4 can be found in human placenta at the mRNA (Figure 6A,C) and protein level (Figure 6B, D). Interestingly, the expression of both syndecan proteins was diminished in placentas from patients suffering from pre-eclampsia as compared to placentas from patients with normally developing pregnancies (Figure 6). The presence of pleiotrophin specifically in human placenta has been reported earlier [35].

### Y-P30 is released from HEK-293 T and COS-7 cells together with the signaling peptide after cleavage from dermcidin

The data so far support a synthesis of Y-P30/dermcidin in placenta. Similar to sweat glands, the peptides are presumably secreted because both act on cells from the outside. Y-P30 triggers signaling at the level of the neuronal membrane and the antimicrobial dermcidin peptides kills bacteria by forming large ion pores in the cell wall [36]. The nature of the secreted peptide species and the mechanism of processing of the precursor are currently only known in sweat glands for the dermcidin [37,38]. We therefore aimed to understand how Y-P30 is secreted from cells. To this end, we generated GFP-fusion constructs containing either the whole precursor protein containing the signal peptide, Y-P30 and dermcidin, only Y-P30 and dermcidin, Y-P30 and the signal peptide, or Y-P30 alone; all of sequences were tagged with GFP (Figure 2A). These constructs were transfected into COS-7 and HEK-293 T cells; both are primate cell lines. The presence of the various peptide fragments was analyzed by employing an antibody that recognizes Y-P30 or by an antibody against the GFP tag in lysates of either the cell pellet or the cell culture supernatant. We found that the Y-P30 portion of the precursor becomes secreted from COS-7 and HEK-293 T cells (Figure 2). The presence of the signal peptide is essential for secretion and intracellular cleavage removes the dermcidin part from the precursor molecule (Figure 2B).

### No evidence for uptake of the Y-P30/dermcidin precursor

**Figure 7:**
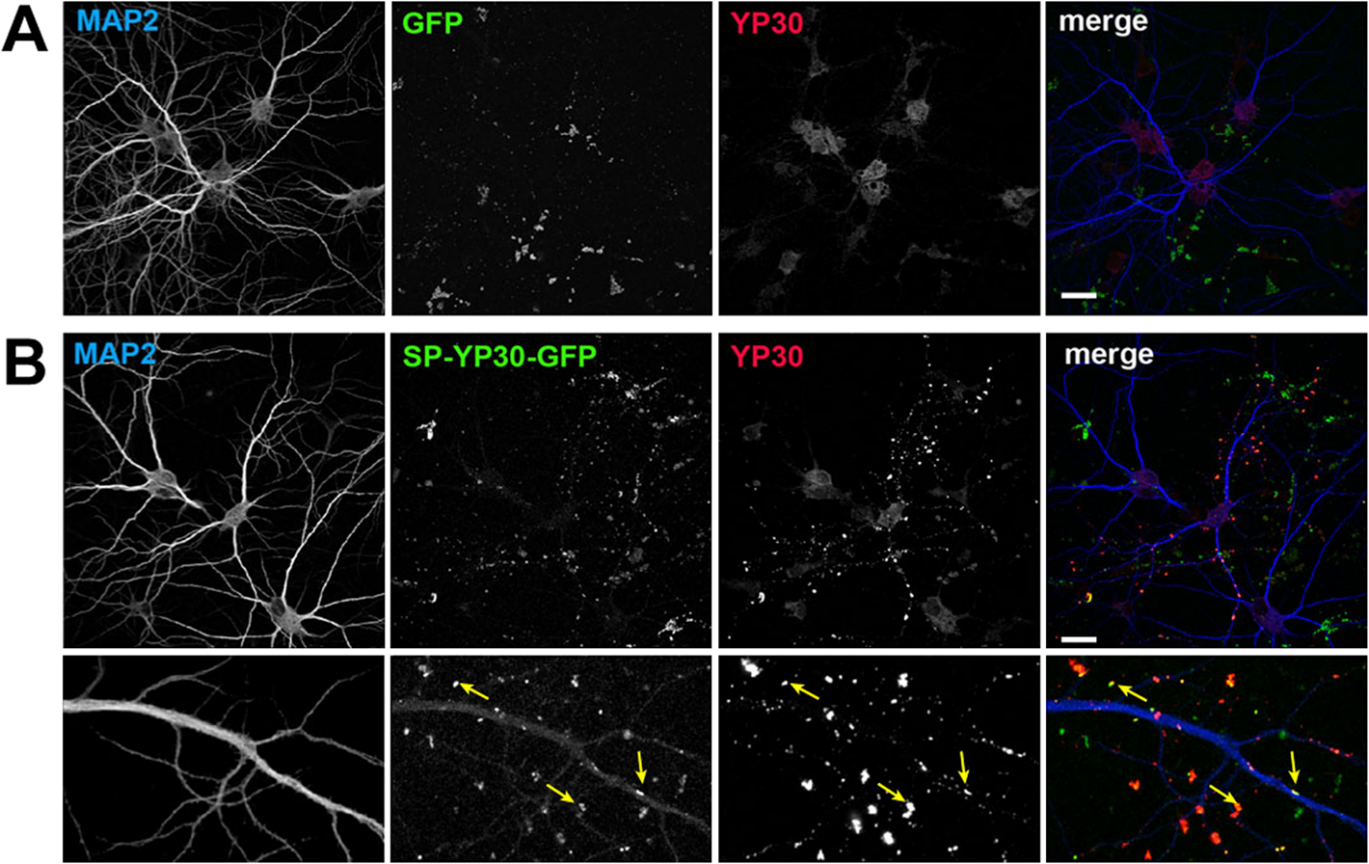
**Administration of bacterially produced and purified GFP (A) or SP-Y-P30-GFP (B) protein to cortical primary neurons does not result in uptake of the proteins.** Cells were stained with a anti-MAP2 antibody to visualize dendritic processes, and with anti-Y-P30 antibody to detect the peptide. Note in **(B)** that SP-Y-P30-GFP deposits are always close to neuronal membranes. Scale bars are 20 μm.

**Figure 8:**
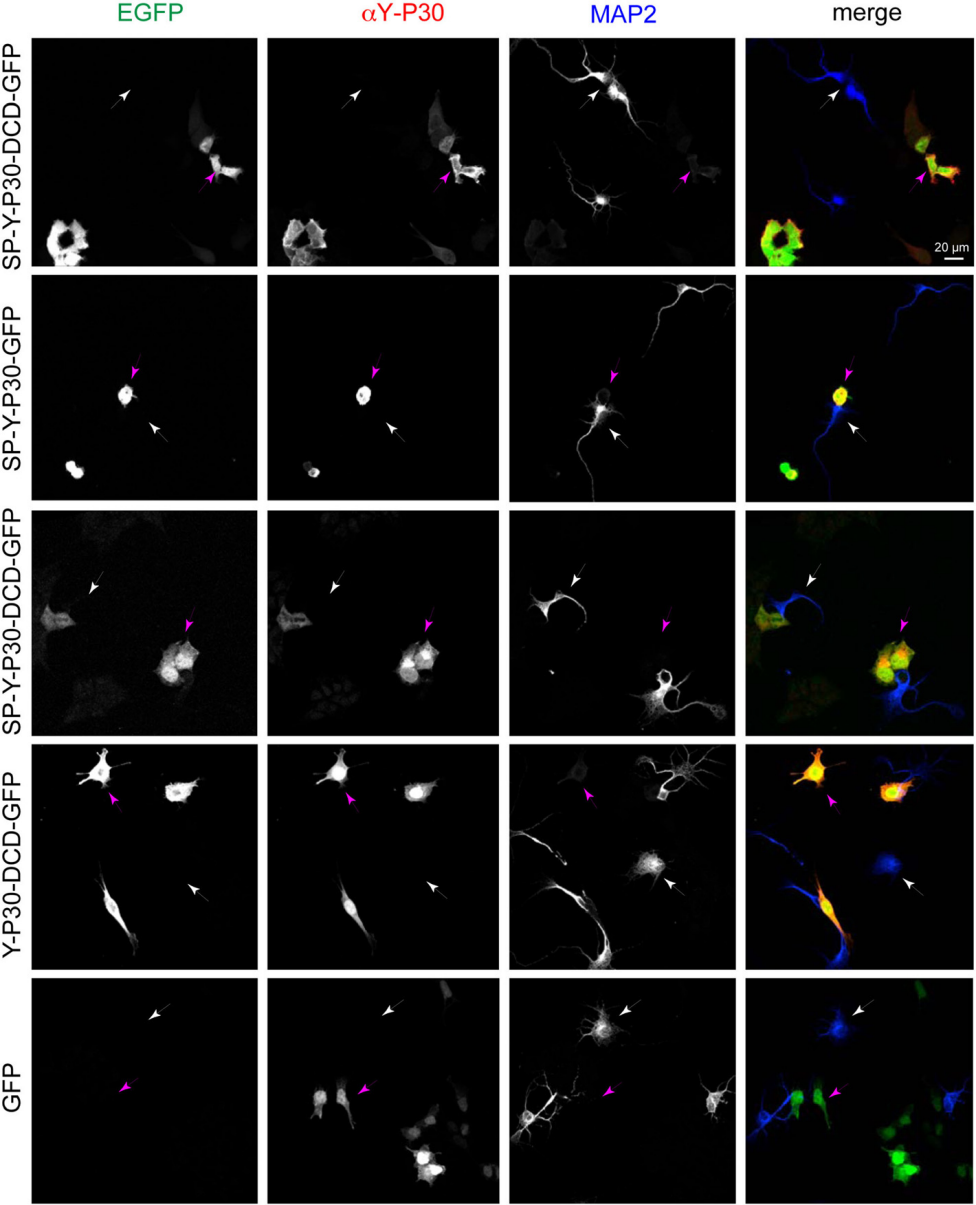
**HEK-293 T cells were transfected with different Y-P30-GFP constructs and co-cultured with cortical primary neurons.** Of the constructs tested, SP-Y-P30-GFP has been shown in Figure 2 to deliver a protein detectable in the culture supernatant. However, the peptide is not taken up into neurons. White arrows point to MAP-2 immunoreactive neurons (blue in the merged picture), purple arrows to transfected HEK-293 T cells (green or red + green in the merged pictures). Scale bar is 20 μm.

Next, we produced and purified large amounts of the full-length Y-P30/dermcidin GFP- tagged precursor containing the signal peptide using the Intein expression system [25]. We applied the purified protein at 10 μg/ml medium to differentiated primary cortical neurons, and we found the GFP-fluorescence aggregating at dendritic sites and a protrusions that resemble spines. However, no uptake into the neurons was apparent (Figure 7).In a co-culture model where we expressed the signal peptide containing fusion protein SP-Y-P30-GFP in HEK-293 T cells, and added these cells to young cortical primary neurons, we found that although the SP-Y-P30-GFP protein is secreted from the HEK-293 T cells (see Figure 2) no uptake of the peptide into the co-cultured neuronal somata or neurites was visible (Figure 8). Taken together these data indicate that fragments of the precursor that contain the signal peptide are not taken up by neurons.

## Discussion and conclusions

In this study we followed up on previous work on the expression and biophysical properties of the survival-promoting peptide Y-P30. A presence of the Y-P30 peptide in non-human primates has been reported in proteomic studies as well as by other methods [1,13,14,23,24,39,40]. However, we and others found that the peptide is extremely stable and in conjunction with the stable mRNA [31], a serious pitfall is a contamination of the samples. Particularly, previous results on the localization of Y-P30 in rodents are erroneous, because the dermcidin gene has not been identified in rodents [12,17]. Given that the Y-P30/dermcidin precursor is present in skin, sweat [12] and tears [41] and probably breath [42,43] the likelihood of contamination is relatively high. Moreover, antibodies might cross-react with remotely related peptides in rodents. We therefore wondered how these findings relate to previous data that suggest expression of Y-P30/dermcidin mRNA in PBMCs of humans and rodents during pregnancy [1]. In a larger number of human samples we could not replicate these previous observations; rather, we found that the presence of the transcript in PBMCs is variable, and is not related to pregnancy. Moreover, application of hormones circulating during pregnancy failed to induce expression of the Y-P30/dermcidin precursor. Although the problems encountered with the detection of the Y-P30/dermcidin peptides and mRNA outlined above make it difficult to come to a final conclusion, it seems unlikely that PBMCs are a source of the Y-P30/dermcidin precursor.

In accord to a previous study [20] we detected Y-P30/dermcidin immunoreactivity in human placenta with immunoblot analysis and ELISA. Dermcidin and Y-P30 have reportedly substantial proteolytical activity [13,20], and dermcidin kills bacteria. Therefore, dermcidin could have local antibiotic effects and protect from infections. Intriguingly, a recent study finds a unique microbiome in the human placenta which has been so far considered to be sterile [44]. Previous studies as well as the present work raise the possibility that Y-P30 is expressed in the placenta. Y-P30 might reach the fetus and the fetal brain to exert neuritogenic activity during development. Indeed, when injecting a bacterially produced tagged Y-P30 peptide into the maternal circulation, the peptide can be detected (using anti-tag antibodies) in brain of the offspring [2]. We now found that the Y-P30 precursor peptide becomes released from overexpressing non-neural cells. When the exogenous peptide was added to differentiated neurons, a binding to dendritic sites resembling spines or spine synapses was observed. Likely this is due to its affinity for pleiotrophin and syndecans [2], and the latter trigger the signaling leading to neuritogenic activities. However, no uptake was observed. Similarly, in the co-culture experiments we could not detect an uptake into young neurons in short term cultures. There was also no aggregation of the peptide to the neuronal membranes which might be due to the immaturity of the neurons employed for this assay. Whether the precursor can be cleaved further by extracellular proteases and whether shorter fragments of Y-P30 peptide could be taken up by neurons is at present unclear.

Y-P30 could also act within the placenta or the trophoblast. The syndecan binding proteins of Y-P30 are present in the placenta. The placental syndecan expression levels were lower in patients suffering from pre-eclampsia. Of note, syndecan-1 expression was already previously suggested as a risk factor for pre-eclampsia [45]. Pleiotrophin and syndecans might regulate trophoblast life cycle and angiogenesis, and intriguingly, the trophoblast expression of pleiotrophin occurs only in human and higher primates [32,35]. Signaling of the primate-specific Y-P30 peptide might be involved in these processes.

## Competing interests

The authors declare that they have no competing interests.

## Authors’ contributions

Performed experiments MM, AS, CB, JN, TE. Analyzed data: MM, AS, TM, PW, MRK. Provide reagents/material PW, ACZ. Designed experiments MM, MRK, ACZ. Wrote the paper MM, ACZ, PW, MRK. All authors read and approved the final version of the manuscript.

## References

[B1] Landgraf P Sieg F Meyer G Wahle P Kreutz MR Pape HC A maternal blood-borne factor promotes survival of the developing thalamus FASEB J 2005 19 225 227 15583035 10.1096/fj.04-1789fje

[B2] Landgraf P Wahle P Pape HC Gundelfinger ED Kreutz MR The survival promoting peptide Y-P30 enhances binding of pleiotrophin to syndecan-2 and -3 and supports its neuritogenic activity J Biol Chem 2008 283 25036 25045 18599487 10.1074/jbc.M800963200 PMC3259816

[B3] Cunningham TJ Hodge L Speicher D Reim D Tyler-Polsz C Levitt P Eagleson K Kennedy S Wang Y Identification of a survival-promoting peptide in medium conditioned by oxidatively stressed cell lines of nervous system origin J Neurosci 1998 18 7047 7060 9736629 10.1523/JNEUROSCI.18-18-07047.1998 PMC6793258

[B4] Cunningham TJ Jing H Wang Y Hodge L Calreticulin binding and other biological activities of survival peptide Y-P30 including effects of systemic treatment of rats Exp Neurol 2000 163 457 468 10833321 10.1006/exnr.2000.7390

[B5] Schneeberg J Riek-Burchardt M Braun H Landgraf P Kreutz MR Reymann KG Neuroprotective effects of the survival promoting peptide Y-P30 Eur J Pharmacol 2009 616 81 85 19490911 10.1016/j.ejphar.2009.05.029

[B6] Macharadze T Landgraf P Pape HC Wahle P Kreutz MR Y-P30 confers neuroprotection after optic nerve crush in adult rats NeuroReport 2011 22 544 547 21666514 10.1097/WNR.0b013e328348b512

[B7] Dash-Wagh S Neumann JR Veitinger S Grote-Westrick C Landgraf P Pape HC Kreutz MR von Holst A Wahle P The survival promoting peptide Y-P30 promotes cellular migration Mol Cell Neurosci 2011 48 195 204 21820515 10.1016/j.mcn.2011.07.005

[B8] Neumann JR Dash-Wagh S Jüngling K Tsai T Meschkat M Räk A Schönfelder S Riedel C Hamad MI Wiese S Pape HC Gottmann K Kreutz MR Wahle P Y-P30 promotes axonal growth by stabilizing growth cones Brain Struct Funct 2014 epub ahead Apr 13 10.1007/s00429-014-0764-2 24728870

[B9] Landgraf P Mikhaylova M Macharadze T Borutzki C Zenclussen AC Wahle P Kreutz MR Binding of Y-P30 to syndecan 2/3 regulates the nuclear localization of CASK PLoS One 2014 9 e85924 24498267 10.1371/journal.pone.0085924 PMC3911912

[B10] Stocki P Wang XN Morris NJ Dickinson AM HSP70 natively and specifically associates with an N-terminal Dermcidin-derived peptide that contains an HLA-A*03 antigenic epitope J Biol Chem 2011 286 12803 12811 21216960 10.1074/jbc.M110.179630 PMC3069480

[B11] Shen SL Qiu FH Dayarathna TK Wu J Kuang M Li SS Peng BG Nie J Identification of Dermcidin as a novel binding protein of Nck1 and characterization of its role in promoting cell migration Biochim Biophys Acta 1812 2011 703 710 10.1016/j.bbadis.2011.03.004 21397687

[B12] Schittek B Hipfel R Sauer B Bauer J Kalbacher H Stevanovic S Schirle M Schroeder K Blin N Meier F Rassner G Garbe C Dermcidin: a novel human antibiotic peptide secreted by sweat glands Nat Immunol 2001 2 1133 1137 11694882 10.1038/ni732

[B13] Todorov P Cariuk P McDevitt T Coles B Fearon K Tisdale M Characterization of a cancer cachectic factor Nature 1996 379 739 742 8602222 10.1038/379739a0

[B14] Monitto CL Dong SM Jen J Sidransky D Characterization of a human homologue of proteolysis-inducing factor and its role in cancer cachexia Clin Cancer Res 2004 10 5862 5869 15355918 10.1158/1078-0432.CCR-04-0435

[B15] Wieland BM Stewart GD Skipworth RJ Sangster K Fearon KC Ross JA Reiman TJ Easaw J Mourtzakis M Kumar V Pak BJ Calder K Filippatos G Kremastinos DT Palcic M Baracos VE Is there a human homologue to the murine proteolysis-inducing factor? Clin Cancer Res 2007 13 4984 4992 17785548 10.1158/1078-0432.CCR-07-0946

[B16] Toll-Riera M Castelo R Bellora N Albà MM Evolution of primate orphan proteins Biochem Soc Trans 2009 37 778 782 19614593 10.1042/BST0370778

[B17] Schittek B The multiple facets of dermcidin in cell survival and host defense J Innate Immun 2012 4 349 360 22455996 10.1159/000336844 PMC6741627

[B18] Ghosh R Maji UK Bhattacharya R Sinha AK The role of dermcidin isoform 2: a two-faceted atherosclerotic risk factor for coronary artery disease and the effect of acetyl salicylic acid on it Thrombosis 2012 2012 987932 22448321 10.1155/2012/987932 PMC3289859

[B19] Ghosh R Jana P Sinha AK The control of hyperglycemia in alloxan treated diabetic mice through the stimulation of hepatic insulin synthesis due to the production of nitric oxide Exp Clin Endocrinol Diabetes 2012 120 145 151 22231923 10.1055/s-0031-1291298

[B20] Lee Motoyama JP Kim-Motoyama H Kim P Nakagama H Miyagawa K Suzuki K Identification of dermcidin in human gestational tissue and characterization of its proteolytic activity Biochem Biophys Res Commun 2007 357 828 833 17448443 10.1016/j.bbrc.2007.03.112

[B21] Porter D Weremowicz S Chin K Seth P Keshaviah A Lahti-Domenici J Bae YK Monitto CL Merlos-Suarez A Chan J Hulette CM Richardson A Morton CC Marks J Duyao M Hruban R Gabrielson E Gelman R Polyak K A neural survival factor is a candidate oncogene in breast cancer Proc Natl Acad Sci U S A 2003 100 10931 10936 12953101 10.1073/pnas.1932980100 PMC196905

[B22] Stewart GD Skipworth RJ Pennington CJ Lowrie AG Deans DA Edwards DR Habib FK Riddick AC Fearon KC Ross JA Variation in dermcidin expression in a range of primary human tumours and in hypoxic/oxidatively stressed human cell lines Br J Cancer 2008 99 126 132 18594538 10.1038/sj.bjc.6604458 PMC2453008

[B23] Park SY Phark S Lee M Zheng Z Choi S Won NH Jung WW Sul D Evaluation of plasma carcinogenic markers in rat hepatic tumors models induced by rat hepatoma N1-S1 cells and benzo[a]pyrene Arch Pharm Res 2010 33 247 255 20195826 10.1007/s12272-010-0210-9

[B24] Yano CL Ventrucci G Field WN Tisdale MJ Gomes-Marcondes MC Metabolic and morphological alterations induced by proteolysis-inducing factor from Walker tumour-bearing rats in C2C12 myotubes BMC Cancer 2008 8 24 18226207 10.1186/1471-2407-8-24 PMC2266935

[B25] Hong I Kim YS Choi SG Simple purification of human antimicrobial peptide dermcidin (MDCD-1 L) by intein-mediated expression in E.coli J Microbiol Biotechnol 2010 20 350 355 20208440

[B26] Hradsky J Raghuram V Reddy PP Navarro G Hupe M Casado V McCormick PJ Sharma Y Kreutz MR Mikhaylova M Post-translational membrane insertion of tail-anchored transmembrane EF-hand Ca2+ sensor calneurons requires the TRC40/Asna1 protein chaperone J Biol Chem 2011 286 36762 36776 21878631 10.1074/jbc.M111.280339 PMC3196075

[B27] Karpova A Mikhaylova M Bera S Bär J Reddy PP Behnisch T Rankovic V Spilker C Bethge P Sahin J Kaushik R Zuschratter W Kähne T Naumann M Gundelfinger ED Kreutz MR Encoding and transducing the synaptic or extrasynaptic origin of NMDA receptor signals to the nucleus Cell 2013 152 1119 1133 23452857 10.1016/j.cell.2013.02.002

[B28] Mikhaylova M Reddy PP Munsch T Landgraf P Suman SK Smalla KH Gundelfinger ED Sharma Y Kreutz MR Calneurons provide a calcium threshold for trans-Golgi network to plasma membrane trafficking Proc Natl Acad Sci U S A 2009 106 9093 9098 19458041 10.1073/pnas.0903001106 PMC2690001

[B29] Zenclussen AC Gerlof K Zenclussen ML Sollwedel A Bertoja AZ Ritter T Kotsch K Leber J Volk HD Abnormal T-cell reactivity against paternal antigens in spontaneous abortion: adoptive transfer of pregnancy-induced CD4 + CD25+ T regulatory cells prevents fetal rejection in a murine abortion model Am J Pathol 2005 166 811 822 15743793 10.1016/S0002-9440(10)62302-4 PMC1602357

[B30] El-Mousleh T Casalis PA Wollenberg I Zenclussen ML Volk HD Langwisch S Jensen F Zenclussen AC Exploring the potential of low doses carbon monoxide as therapy in pregnancy complications Med Gas Res 2012 2 4 22348450 10.1186/2045-9912-2-4 PMC3837472

[B31] Sakurada K Akutsu T Fukushima H Watanabe K Yoshino M Detection of dermcidin for sweat identification by real-time RT-PCR and ELISA Forensic Sci Int 2010 194 80 84 19914015 10.1016/j.forsciint.2009.10.015

[B32] Chui A Zainuddin N Rajaraman G Murthi P Brennecke SP Ignjatovic V Monagle PT Said JM Placental syndecan expression is altered in human idiopathic fetal growth restriction Am J Pathol 2012 180 693 702 22138583 10.1016/j.ajpath.2011.10.023

[B33] Drapel V Becue A Champod C Margot P Identification of promising antigenic components in latent fingermark residues Forensic Sci Int 2009 184 47 53 19147311 10.1016/j.forsciint.2008.11.017

[B34] Pathak S De Souza GA Salte T Wiker HG Asjö B HIV induces both a down-regulation of IRAK-4 that impairs TLR signalling and an up-regulation of the antibiotic peptide dermcidin in monocytic cells Scand J Immunol 2009 70 264 276 19703016 10.1111/j.1365-3083.2009.02299.x

[B35] Ball M Carmody M Wynne F Dockery P Aigner A Cameron I Higgins J Smith SD Aplin JD Moore T Expression of pleiotrophin and its receptors in human placenta suggests roles in trophoblast life cycle and angiogenesis Placenta 2009 30 649 653 19481257 10.1016/j.placenta.2009.05.001

[B36] Song C Weichbrodt C Salnikov ES Dynowski M Forsberg BO Bechinger B Steinem C de Groot BL Zachariae U Zeth K Crystal structure and functional mechanism of a human antimicrobial membrane channel Proc Natl Acad Sci U S A 2013 110 4586 4591 23426625 10.1073/pnas.1214739110 PMC3607029

[B37] Baechle D Flad T Cansier A Steffen H Schittek B Tolson J Herrmann T Dihazi H Beck A Mueller GA Mueller M Stevanovic S Garbe C Mueller CA Kalbacher H Cathepsin D is present in human eccrine sweat and involved in the postsecretory processing of the antimicrobial peptide DCD-1 L J Biol Chem 2006 281 5406 5415 16354654 10.1074/jbc.M504670200

[B38] Flad T Bogumil R Tolson J Schittek B Garbe C Deeg M Mueller CA Kalbacher H Detection of dermcidin-derived peptides in sweat by ProteinChip technology J Immunol Methods 2002 270 53 62 12379338 10.1016/s0022-1759(02)00229-6

[B39] Ambatipudi K Joss J Deane E A comparative proteomic analysis of skin secretions of the tammar wallaby (Macropus eugenii) and the wombat (Vombatus ursinus) Comp Biochem Physiol Part D Genomics Proteomics 2007 2 322 331 20483304 10.1016/j.cbd.2007.07.001

[B40] de Lima C Alves LE Iagher F Machado AF Bonatto SJ Kuczera D de Souza CF Pequito DC Muritiba AL Nunes EA Fernandes LC Anaerobic exercise reduces tumor growth, cancer cachexia and increases macrophage and lymphocyte response in Walker 256 tumor-bearing rats Eur J Appl Physiol 2008 104 957 964 18688637 10.1007/s00421-008-0849-9

[B41] You J Fitzgerald A Cozzi PJ Zhao Z Graham P Russell PJ Walsh BJ Willcox M Zhong L Wasinger V Li Y Post-translation modification of proteins in tears Electrophoresis 2010 31 1853 1861 20506419 10.1002/elps.200900755

[B42] Bloemen K Van Den Heuvel R Govarts E Hooyberghs J Nelen V Witters E Desager K Schoeters G A new approach to study exhaled proteins as potential biomarkers for asthma Clin Exp Allergy 2011 41 346 356 21105917 10.1111/j.1365-2222.2010.03638.x

[B43] Chang WC Huang MS Yang CJ Wang WY Lai TC Hsiao M Chen CH Dermcidin identification from exhaled air for lung cancer diagnosis Eur Respir J 2010 35 1182 1185 20436176 10.1183/09031936.00169509

[B44] Aagaard K Ma J Antony KM Ganu R Petrosino J Versalovic J The placenta harbors a unique microbiome Sci Transl Med 2014 6 237ra65 10.1126/scitranslmed.3008599 PMC4929217 24848255

[B45] Schmedt A Götte M Heinig J Kiesel L Klockenbusch W Steinhard J Evaluation of placental syndecan-1 expression in early pregnancy as a predictive fetal factor for pregnancy outcome Prenat Diagn 2012 32 131 137 22418956 10.1002/pd.2908

